# Impact of chronic obstructive pulmonary disease on 10-year mortality after percutaneous coronary intervention and bypass surgery for complex coronary artery disease: insights from the SYNTAX Extended Survival study

**DOI:** 10.1007/s00392-021-01833-y

**Published:** 2021-03-12

**Authors:** Rutao Wang, Mariusz Tomaniak, Kuniaki Takahashi, Chao Gao, Hideyuki Kawashima, Hironori Hara, Masafumi Ono, David van Klaveren, Robert-Jan van Geuns, Marie-Claude Morice, Piroze M. Davierwala, Michael J. Mack, Adam Witkowski, Nick Curzen, Sergio Berti, Francesco Burzotta, Stefan James, Arie Pieter Kappetein, Stuart J. Head, Daniel J. F. M. Thuijs, Friedrich W. Mohr, David R. Holmes, Ling Tao, Yoshinobu Onuma, Patrick W. Serruys

**Affiliations:** 1 Department of Cardiology, Xijing Hospital, Xi’an, China; 2 Department of Cardiology, National University of Ireland, Galway (NUIG), P.O. University Road, Galway, H91 TK33 Ireland; 3 Department of Cardiology, Radboud University Medical Center, Nijmegen, The Netherlands; 4 First Department of Cardiology, Medical University of Warsaw, Warsaw, Poland; 5 Department of Cardiology, Erasmus University Medical Center, Rotterdam, The Netherlands; 6 Department of Cardiology, Amsterdam Universities Medical Centers, Location Academic Medical Center, University of Amsterdam, Amsterdam, The Netherlands; 7 Department of Public Health, Erasmus University Medical Center, Rotterdam, The Netherlands; 8 Predictive Analytics and Comparative Effectiveness Center, Institute for Clinical Research and Health Policy Studies, Tufts Medical Center, Boston, USA; 9 ICPS Ramsay-Generale de Sante, Massy, France; 10 Department of Cardiac Surgery, Heart Centre Leipzig, Leipzig, Germany; 11 Baylor Scott & White Health, Plano, TX USA; 12 Department of Interventional Cardiology and Angiology, National Institute of Cardiology, Warsaw, Poland; 13 Cardiology Department, University Hospital Southampton, Southampton, UK; 14 Cardiology Department, Heart Hospital-Fondazione C.N.R. Reg. Toscana G. Monasterio, Massa, Italy; 15 Institute of Cardiology, Fondazione Policlinico Universitario Agostino Gemelli IRCCS, Università Cattolica del Sacro Cuore, Rome, Italy; 16 Department of Medical Sciences and Uppsala Clinical Research Center, Uppsala University, Uppsala, Sweden; 17 Department of Cardiothoracic Surgery, Erasmus University Medical Centre, Rotterdam, The Netherlands; 18 Mayo Clinic, Rochester, MN USA; 19 NHLI, Imperial College London, London, UK; 20 Erasmus University Medical Center, Rotterdam, The Netherlands

**Keywords:** All-cause death, Chronic obstructive pulmonary disease, Coronary artery bypass grafting, Percutaneous coronary intervention, SYNTAX

## Abstract

**Aims:**

To evaluate the impact of chronic obstructive pulmonary disease (COPD) on 10-year all-cause death and the treatment effect of CABG versus PCI on 10-year all-cause death in patients with three-vessel disease (3VD) and/or left main coronary artery disease (LMCAD) and COPD.

**Methods:**

Patients were stratified according to COPD status and compared with regard to clinical outcomes. Ten-year all-cause death was examined according to the presence of COPD and the revascularization strategy.

**Results:**

COPD status was available for all randomized 1800 patients, of whom, 154 had COPD (8.6%) at the time of randomization. Regardless of the revascularization strategy, patients with COPD had a higher risk of 10-year all-cause death, compared with those without COPD (43.1% vs. 24.9%; hazard ratio [HR]: 2.03; 95% confidence interval [CI]: 1.56–2.64; *p* < 0.001). Among patients with COPD, CABG appeared to have a slightly lower risk of 10-year all-cause death compared with PCI (42.3% vs. 43.9%; HR: 0.96; 95% CI: 0.59–1.56, *p* = 0.858), whereas among those without COPD, CABG had a significantly lower risk of 10-year all-cause death (22.7% vs. 27.1%; HR: 0.81; 95% CI: 0.67–0.99, *p* = 0.041). There was no significant differential treatment effect of CABG versus PCI on 10-year all-cause death between patients with and without COPD (*p*_interaction_ = 0.544).

**Conclusions:**

COPD was associated with a higher risk of 10-year all-cause death after revascularization for complex coronary artery disease. The presence of COPD did not significantly modify the beneficial effect of CABG versus PCI on 10-year all-cause death.

*Trial registration:* SYNTAX: ClinicalTrials.gov reference: NCT00114972. SYNTAX Extended Survival: ClinicalTrials.gov reference: NCT03417050

**Graphic abstract:**

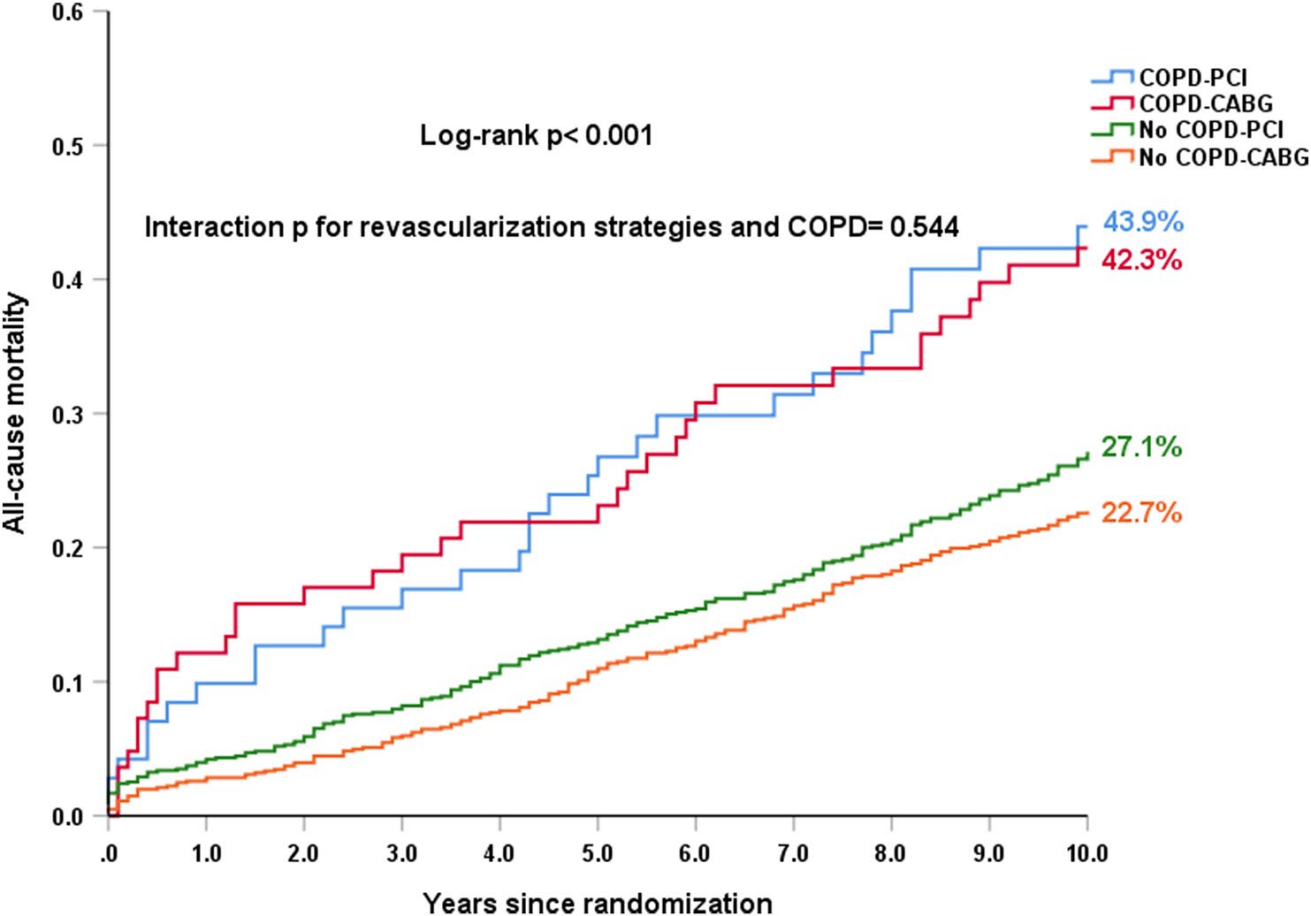

## Introduction

Chronic obstructive pulmonary disease (COPD) is associated with accelerated atherosclerosis and cardiovascular disease; therefore, they frequently coexist [1, 2]. Known as a risk factor for cardiovascular mortality [3], the status of COPD is a variable in the formula to calculate SYNTAX score II [4–6], Southern Thoracic Society (STS) score [7], EuroSCORE II [8], and more recently—the SYNTAX score II 2020 [9]. Patients with COPD are perceived to be at increased surgical risk, and are often referred to percutaneous coronary intervention (PCI) instead of coronary bypass artery grafting (CABG). However, limited data support this preference. Most studies have indeed demonstrated that COPD patients undergoing CABG had increased in-hospital and long-term mortality [10, 11], whilst some studies reported discrepant results [12, 13]. On the other hand, patients with COPD who underwent PCI have worse prognosis compared with those without COPD [14–17]. Furthermore, in the EXCEL (Evaluation of XIENCE Versus Coronary Artery Bypass Surgery for Effectiveness of Left Main Revascularization) trial, COPD was associated with worse clinical outcomes after left main coronary artery disease (LMCAD) revascularization. Yet the relative risks of PCI versus CABG at 30 days and 3 years were similar irrespective of the presence of COPD in this specific subset of patients [18]. Currently, therefore, the optimal revascularization strategy for patients with COPD and complex CAD remains debatable.

The SYNTAX Extended Survival (SYNTAXES) study reported the 10-year all-cause mortality in 94% of all the patients with de novo three-vessel disease (3VD) and/or LMCAD who were originally randomized to CABG or PCI in the SYNTAX trial [19]. In the present study, we aimed 1) to evaluate the impact of COPD on 10-year all-cause mortality and 2) to estimate the treatment effect of CABG versus PCI for 10-year all-cause mortality according to COPD in patients with 3VD and/or LMCAD.

## Methods

### Study design and population

The design and the primary results of the SYNTAX trial have been published elsewhere [20–22]. In brief, the SYNTAX trial (NCT00114972) was an international, multicenter, randomized controlled trial conducted between March 2005 and April 2007. Based on clinical judgment and the consensus of a Heart Team, all-comers patients with de novo 3VD and/or LMCAD deemed eligible for both PCI and CABG were enrolled and randomized in a 1:1 fashion to either CABG (*n* = 897) or PCI (*n* = 903) with the TAXUS Express paclitaxel-drug eluting stents (Boston Scientific Corporation, Marlborough, MA, USA). The trial completed the patient follow-up to 5 years [22]. The SYNTAXES study (NCT03417050) was an investigator-driven initiative that extended follow-up and aimed to evaluate vital status up to 10 years [19]. The longest follow-up was 14.1 years. The extended follow-up study was funded by the German Heart Research Foundation (GHF; Frankfurt am Main, Germany). Follow-up was conducted in accordance with local regulations of each participating center and complied with the declaration of Helsinki.

### Definitions and endpoints

COPD at baseline was defined as long-term use of bronchodilators or steroids for lung disease according to the definition in EuroSCORE [23]. The primary endpoint of the SYNTAXES study was all-cause death at 10 years. The 30-day and 5-year major adverse cardiovascular and cerebrovascular events (MACCE, defined as a composite endpoint of all-cause death, cerebrovascular accident, myocardial infarction [MI] or repeat revascularization, the primary endpoint of the SYNTAX trial) according to the status of COPD were also explored in the current analysis. Vital status was confirmed by contact with medical care personnel and/or by electronic healthcare record review and national death registry.

### Statistical analysis

All the analyses were performed according to the intention to treat principle. Continuous variables are reported as mean ± standard deviations, and were compared using Student’s *t* test or Mann–Whitney *U* test. Categorical variables are shown as percentages and numbers and were compared using Fisher’s exact test. Time-to-event Kaplan–Meier estimates with log-rank test were used to compare COPD versus non-COPD in the PCI and CABG arm, respectively, and to compare PCI with CABG according to COPD. Cox proportional hazards regression was used to calculate hazard ratios (HRs) with 95% confidence interval (CI). Multivariate analysis was performed to investigate whether COPD was an independent predictor of all-cause death at 10 years. The Cox proportional hazards regression model included the following covariates: age, gender, body mass index, current smoking, peripheral vascular disease, left ventricular ejection fraction (LVEF), creatinine clearance (ml/min), prior MI, prior stroke, and the anatomical SYNTAX score, which have been selected based on previous knowledge of the association of those variables with the clinical outcomes [24]. All analyses were performed using SPSS Statistics, version 25 (IBM Corp., Armonk, 281 N.Y., USA) and a *p* value of < 0.05 was considered to be statistically significant.

## Results

### Baseline characteristics

COPD status was available in all patients randomized in the SYNTAX trial. Of the 1800 participants, 154 (8.6%) had COPD. Baseline characteristics according to COPD status are shown in Table 1. Patients with COPD were more likely to be older, had more cardiovascular risk factors (previous carotid artery disease, peripheral vascular disease, congestive heart failure, and current smoking), and had a higher EuroSCORE and Parsonnet SCORE, as compared to those without COPD. They were less likely to receive arterial conduits and to take aspirin and beta blockers at discharge. Baseline clinical and procedural characteristics according to COPD as well as randomized revascularization strategies are reported in Table 2. By randomization, baseline clinical and procedural characteristics were largely well balanced between PCI and CABG in patients with and without COPD.

**Table 1:** Baseline characteristics according to COPD

	COPD (*n* = 154)	No COPD (*n* = 1646)	*p* value
PCI	46.1 (71/154)	50.5 (832/1646)	0.292
CABG	53.9 (83/154)	49.5 (814/1646)	
Age (year)	66.9 ± 8.7	64.9 ± 9.8	0.017
Sex			
Male	73.4 (113/154)	78.1 (1285/1646)	0.181
Body mass index (kg/m^2^)	28.6 ± 5.6	28.0 ± 4.6	0.164
Medically treated diabetes	29.2 (45/154)	24.7 (407/1646)	0.219
On insulin	13 (20/154)	9.8 (162/1646)	0.216
Metabolic syndrome	40.3 (62/154)	36.1 (594/1646)	0.352
Hypertension	71.4 (110/154)	66 (1086/1646)	0.171
Dyslipidemia	77.8 (119/153)	77.9 (1272/1632)	0.963
Current smoker	27.9 (43/154)	19.5 (320/1639)	0.013
Previous MI	36 (54/150)	32.6 (531/1630)	0.393
Previous stroke	5.3 (8/150)	4.3 (70/1639)	0.542
Previous TIA	7.3 (11/151)	4.5 (73/1638)	0.116
Previous carotid artery disease	16.2 (25/154)	7.5 (123/1646)	< 0.001
PVD	19.5 (30/154)	8.9 (147/1646)	< 0.001
Impaired renal function	22.1 (34/154)	17.1 (282/1646)	0.155
Creatinine clearance (ml/min)	84.8 ± 32.9	86.2 ± 32.7	0.630
LVEF (%)	56.9 ± 14.2	58.8 ± 12.9	0.171
Congestive heart failure	8.5 (13/153)	4.3 (70/1625)	0.019
Clinical presentation			0.432
Silent ischemia	14.9 (23/154)	14.4 (237/1646)	
Stable angina	61 (94/154)	56.7 (933/1646)	
Unstable angina	24 (37/154)	28.9 (476/1646)	
Euro SCORE	5.2 ± 2.9	3.6 ± 2.6	< 0.001
Parsonnet SCORE	9.9 ± 6.9	8.4 ± 6.9	0.008
Disease extent			0.956
3VD	60.8 (1001/1646)	61 (94/154)	
LMCAD	39 (60/154)	39.2 (645/1646)	
Disease extent			0.806
LMCAD only	6.5 (10/153)	4.9 (81/1646)	
LMCAD + 1VD	7.8 (12/153)	7.7 (126/1646)	
LMCAD + 2VD	13.7 (21/153)	12 (1646/241)	
LMCAD + 3VD	11.1 (17/153)	14.6 (241/1646)	
2VD	2 (3/153)	2 (33/1646)	
3VD	58.8 (90/153)	58.8 (968/1646)	
Anatomical SYNTAX score	29.6 ± 11.8	28.7 ± 11.4	0.344
Number of lesions	4.5 ± 1.9	4.3 ± 1.8	0.461
Any total occlusion	18.3 (28/153)	23.7 (387/1634)	0.132
Any bifurcation	76.5 (117/153)	72.4 (1183/1634)	0.279
Number of stents	4.6 ± 2.6	4.6 ± 2.2	0.924
TSL per patient	85.5 ± 52.9	85.7 ± 47.5	0.980
Off pump CABG	4.5 (7/154)	7.5 (123/1646)	0.215
LIMA use	44.2 (68/154)	41.1 (676/1646)	0.757
Number of total conduits	2.7 ± 0.8	2.8 ± 0.7	0.754
Number of arterial conduits	1.2 ± 0.5	1.4 ± 0.7	0.001
Number of venous conduits	1.5 ± 0.9	1.3 ± 0.9	0.103
Complete revascularization	61.6 (93/151)	59.8 (965/1615)	0.660
Medication at discharge			
Aspirin	87.4 (132/151)	92.9 (1501/1615)	0.014
Thienopyridine	53.6 (81/151)	59.2 (956/1615)	0.185
Statin	78.8 (119/151)	80.9 (1306/1615)	0.540
Beta blockers	60.3 (91/151)	81.8 (1321/1615)	< 0.001
ACEI	47.7 (72/151)	50.2 (810/1615)	0.561
ARB	14.6 (22/151)	9.8 (158/1615)	0.063

*ACEI* angiotensin-converting enzyme inhibitors, *ARB* angiotensin II receptor blockers, *CABG* coronary bypass artery grafting, *LMCAD* left main coronary artery disease, *LVEF* left ventricular ejection fraction, *MI* myocardial infarction, *PCI* percutaneous coronary intervention, *PVD* peripheral vascular disease, *TIA* transient ischemia attack, *TSL* total stent length, *3VD* three-vessel disease

**Table 2:** Baseline characteristics according to COPD and revascularization strategies

	COPD (*N* = 154)		*p* value	No COPD (*N* = 1646)		*p* value
	PCI (*N* = 71)	CABG (*N* = 83)		PCI (*N* = 832)	CABG (*N* = 814)	
Age (year)	66.8 ± 8.9	67 ± 8.5	0.916	65.1 ± 9.7	64.8 ± 9.9	0.476
Sex						
Male	71.8 (51/71)	74.7 (62/83)	0.688	76.8 (639/832)	79.4 (646/814)	0.210
Body mass index (kg/m^2^)	29.3 ± 5.5	28 ± 5.6	0.147	28 ± 4.7	27.9 ± 4.4	0.648
Medically treated diabetes	33.8 (24/71)	25.3 (21/83)	0.248	24.9 (207/832)	24.6 (200/814)	0.884
On insulin	14.1 (10/71)	12 (10/83)	0.708	9.5 (79/832)	10.2 (83/814)	0.633
Metabolic syndrome	46.5 (33/71)	34.9 (29/83)	0.032	36.8 (306/832)	35.4 (288/814)	0.380
Hypertension	74.6 (53/71)	68.7 (57/83)	0.413	68.4 (569/832)	63.5 (517/814)	0.037
Dyslipidemia	76.1 (54/71)	79.3 (65/82)	0.634	78.9 (651/825)	77 (621/807)	0.340
Current smoker	25.4 (18/71)	30.1 (25/83)	0.511	17.9 (149/832)	21.2 (171/807)	0.094
Previous MI	33.3 (23/69)	38.3 (31/81)	0.530	31.8 (262/824)	33.4 (269/806)	0.497
Previous stroke	7.2 (5/69)	3.7 (3/81)	0.336	3.6 (30/830)	4.9 (40/809)	0.183
Previous TIA	10 (7/70)	4.9 (4/81)	0.233	3.9 (32/831)	5.1 (41/807)	0.228
Previous carotid artery disease	19.7 (14/71)	13.3 (11/83)	0.278	7.1 (59/832)	7.9 (64/814)	0.552
PVD	21.1 (15/71)	18.1 (15/83)	0.633	8.1 (67/832)	9.8 (80/814)	0.207
Impaired renal function	23.9 (17/71)	20.5 (17/83)	0.139	18 (150/832)	16.2 (132/814)	< 0.001
Creatinine clearance (ml/min)	86.2 ± 34.7	83.6 ± 31.3	0.650	86.7 ± 35.6	85.7 ± 29.3	0.565
LVEF (%)	58.2 ± 12.3	55.6 ± 15.9	0.363	59.1 ± 13	58.5 ± 12.9	0.453
Congestive heart failure	4.2 (3/71)	12.2 (10/82)	0.078	4 (33/827)	4.6 (37/798)	0.521
Clinical presentation			0.138			0.998
Silent ischemia	9.9 (7/71)	19.3 (16/83)		14.4 (120/832)	14.4 (117/814)	
Stable angina	60.6 (43/71)	61.4 (51/83)		56.6 (471/832)	56.8 (462/814)	
Unstable angina	29.6 (21/71)	19.3 (16/83)		29 (241/832)	28.9 (235/814)	
Euro SCORE	5.4 ± 3	5.1 ± 2.7	0.636	3.6 ± 2.5	3.6 ± 2.7	0.802
Parsonnet SCORE	10.5 ± 7.4	9.4 ± 6.5	0.343	8.4 ± 6.9	8.3 ± 6.9	0.924
Disease extent			0.657			0.842
3VD	59.2 (42/71)	62.7 (52/83)		60.6 (504/832)	61.1 (497/814)	
LMCAD	40.8 (29/71)	37.3 (31/83)		39.4 (328/832)	38.9 (317/814)	
Disease extent			0.983			0.915
LMCAD only	7 (5/71)	6.1 (5/82)		4.4 (37/832)	5.4 (44/814)	
LMCAD + 1VD	7 (5/71)	8.5 (7/82)		7.5 (62/832)	7.9 (64/814)	
LMCAD + 2VD	14.1 (71/9)	13.4 (11/82)		12.3 (832/127)	11.7 (95/814)	
LMCAD + 3VD	12.7 (9/71)	9.8 (8/82)		15.3 (127/832)	14 (114/814)	
2VD	1.4 (1/71)	2.4 (2/82)		1.9 (16/832)	2.1 (17/814)	
3VD	57.7 (41/71)	59.8 (49/82)		58.7 (488/832)	59 (480/814)	
Anatomical SYNTAX score	30 ± 12.6	29.2 ± 11.1	0.666	28.3 ± 11.4	29.1 ± 11.4	0.149
Number of lesions	4.5 ± 2	4.4 ± 1.8	0.643	4.3 ± 1.8	4.4 ± 1.8	0.426
Any total occlusion	25.4 (18/71)	12.2 (10/82)	0.036	24.1 (199/826)	23.3 (188/808)	0.695
Any bifurcation	74.6 (53/71)	78 (64/82)	0.621	72.2 (596/826)	72.6 (587/808)	0.823
Number of stents	4.7 ± 2.6	–		4.6 ± 2.2	–	
TSL per patient	86.6 ± 53.2	–		86.4 ± 47.5	–	
Off pump CABG	–	8.4 (7/83)		–	14.9 (121/814)	
LIMA use	–	81.9 (68/83)		–	81.8 (666/814)	
Number of total conduits	–	2.7 ± 0.8		–	–	0.300
Number of arterial conduits	–	1.2 ± 0.5		–	–	0.005
Number of venous conduits	–	1.5 ± 0.9		–	–	0.700
Complete revascularization	53.5 (38/71)	68.8 (55/80)	0.055	57 (470/825)	62.7 (495/790)	0.020
Medication at discharge						
Aspirin	93 (66/71)	82.5 (66/80)	0.053	96.6 (797/825)	89.1 (704/790)	< 0.001
Thienopyridine	94.4 (67/71)	17.5 (14/80)	< 0.001	97 (800/825)	19.7 (156/790)	< 0.001
Statin	84.5 (60/71)	73.8 (59/80)	0.106	86.9 (717/825)	74.6 (589/790)	< 0.001
Beta blockers	56.3 (40/71)	63.8 (51/80)	0.353	83.4 (688/825)	80.1 (633/790)	0.089
ACEI	49.3 (35/71)	46.3 (37/80)	0.708	55.6 (459/825)	44.4 (351/790)	< 0.001
ARB	21.1 (15/71)	8.8 (7/80)	0.031	12.6 (104/825)	6.8 (54/790)	< 0.001

*ACEI* angiotensin-converting enzyme inhibitors, *ARB* angiotensin II receptor blockers, *CABG* coronary bypass artery grafting, *LMCAD* left main coronary artery disease, *LVEF* left ventricular ejection fraction, *MI* myocardial infarction, *PCI* percutaneous coronary intervention, *PVD* peripheral vascular disease, *TIA* transient ischemia attack, *TSL* total stent length, *3VD* three-vessel disease

### Outcomes according to COPD

**Fig. 1:**
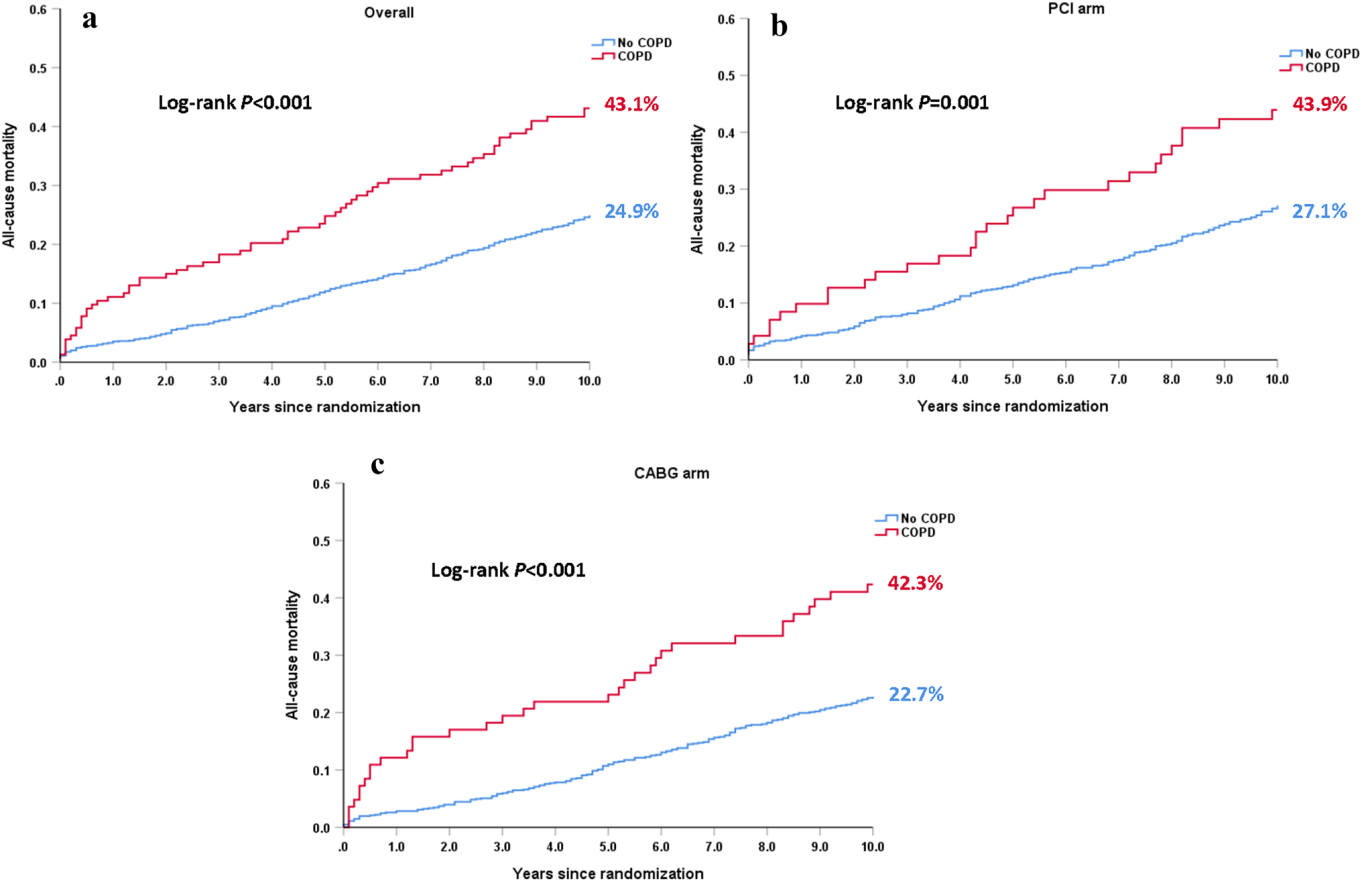
Kaplan–Meier curves for all-cause death at 10 years in patients with (red) or without (blue) COPD among the overall cohort, the PCI arm and the CABG arm. **a** 10-year all-cause mortality according to COPD in the overall cohort. **b** 10-year all-cause mortality according to COPD in the PCI arm. **c** 10-year all-cause mortality according to COPD in the CABG arm. Event rates represent Kaplan–Meier estimates

When compared to those without COPD, patients with COPD had a comparable MACCE rate at 30 days (5.8% vs. 5.2%, HR: 1.12, 95% CI: 0.57–2.24, *p* = 0.738), but had a higher 5-year MACCE rate (41.4% vs. 32.1%, HR: 1.42, 95% CI: 1.09–1.85, *p* = 0.010), which was mainly driven by a higher 5-year all-cause death (23.2% vs. 12.2%, HR: 2.15, 95% CI: 1.49–3.10, *p* < 0.001) (Table 3). A significantly higher risk of all-cause death at 10 years was observed in the patients with COPD, compared with those without COPD (43.1% vs. 24.9%; HR: 2.03; 95% CI: 1.56–2.64; *p* < 0.001, Fig. 1a, Table 3). COPD was associated with a higher 10-year all-cause death in both PCI and CABG arms (Fig. 1b, c).

**Table 3:** Risk of COPD on outcomes according to treatment strategies

	COPD	No COPD	Unadjusted HR (95% CI)	*p* value
	(*n* = 154)	(*n* = 1646)		
*At 30 days*				
MACCE	5.8 (9)	5.2 (85)	1.12 (0.57–2.24)	0.738
Death, stroke, MI	5.8 (9)	4.1 (67)	1.43 (0.71–2.87)	0.311
All-cause death	3.3 (5)	1.2 (20)	2.66 (1.00–7.09)	0.050
Cardiac death	3.3 (5)	1.2 (20)	2.66 (1.00–7.09)	0.050
Any MI	4.6 (7)	2.9 (48)	1.56 (0.71–3.45)	0.272
Any stroke	0 (0)	0.7 (12)	0.04 (0–314.05)	0.489
Any repeat revascularization	2.0 (3)	2.3 (37)	0.86 (0.27–2.8)	0.806
*At 5 years*				
MACCE	41.4 (61)	32.1 (500)	1.42 (1.09–1.85)	0.010
Death, stroke, MI	29.9 (44)	18.3 (284)	1.81 (1.32–2.49)	< 0.001
All-cause death	23.2 (34)	12.2 (186)	2.15 (1.49–3.10)	< 0.001
Cardiac death	13.8 (20)	6.7 (102)	2.27 (1.41–3.67)	0.001
Any MI	10.0 (14)	6.6 (102)	1.56 (0.89–2.73)	0.119
Any stroke	4.4 (6)	2.9 (45)	1.53 (0.65–3.58)	0.329
Any repeat revascularization	22.1 (29)	20.2 (303)	1.11 (0.76–1.63)	0.583
*At 10 years*				
All-cause death	43.1 (64)	24.9 (396)	2.03 (1.56–2.64)	< 0.001

*COPD* chronic obstructive pulmonary disease, *MI* myocardial infarction, *MACCE* major adverse cardiovascular and cerebrovascular event. Data are presented as percentage based on Kaplan–Meier estimates (number of deaths)

### Clinical outcomes according to revascularization strategy

**Fig. 2:**
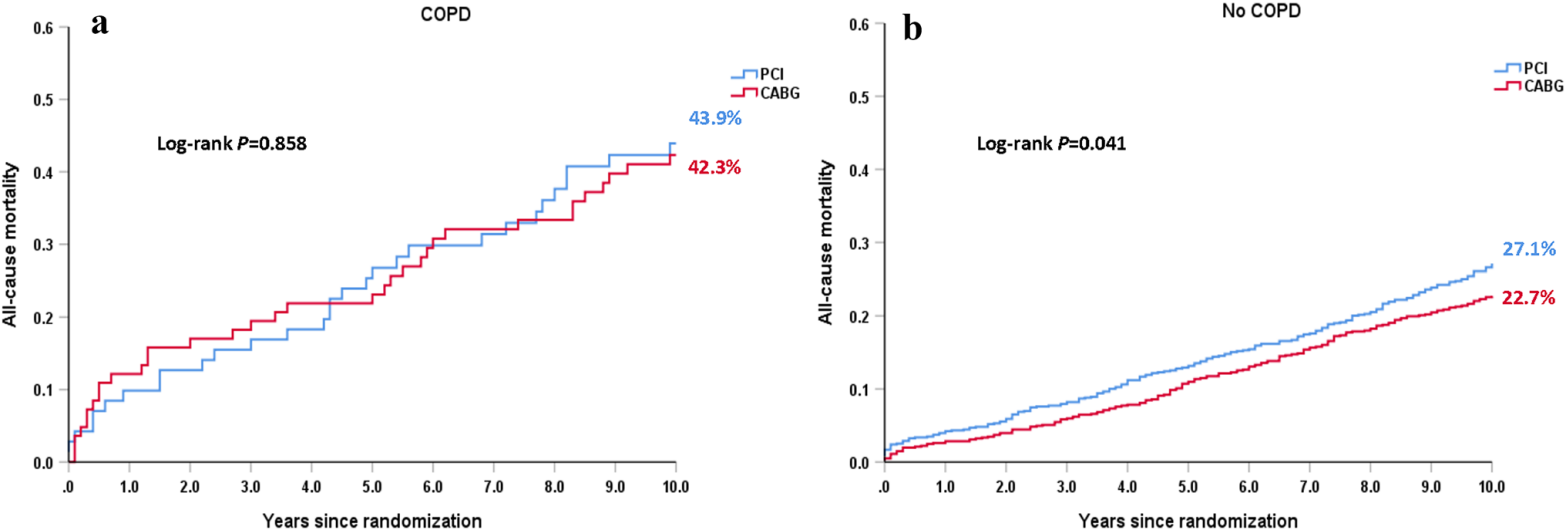
Kaplan–Meier curves for all-cause death at 10 years in patients randomized to PCI (blue) vs. CABG (red) among patients with and without COPD. **a** All-cause mortality at 10 years in patients with COPD. **b** All-cause mortality at 10 years in patients without COPD. Event rates represent Kaplan–Meier estimates

There were 71 and 83 COPD patients randomized to PCI and CABG, respectively. Among these patients, differences between CABG and PCI for 30-day MACCE (4.8% vs. 7.0%, HR: 0.66, 95% CI: 0.18–2.46, *p* = 0.538) and 5-year MACCE (35.9% vs. 47.3%, HR: 0.73, 95% CI: 0.44–1.2, *p* = 0.213) were not statistically significant (Table 4). At 10 years, CABG appeared to have a slightly lower risk of all-cause death compared with PCI (42.3% vs. 43.9%; HR: 0.96, 95% CI: 0.59–1.56, *p* = 0.858, Fig. 2a, Table 4) in patients with COPD. By contrast, among those without COPD, CABG had a significantly lower risk of 10-year all-cause death (22.7% vs. 27.1%; HR: 0.81, 95% CI: 0.67–0.99, *p* = 0.041, Fig. 2b, Table 4). There was no significant differential treatment effect of CABG versus PCI on the 10-year all-cause death for patients without COPD and patients with COPD (*p*_interaction_ = 0.544, Table 4).

**Table 4:** Treatment effect on outcomes in COPD and non-COPD patients

	COPD (*N* = 154)			*p* value	No COPD (*N* = 1646)			*p* value	*p* interaction
	CABG (*N* = 83)	PCI (*N* = 71)	HR (95% CI)		CABG (*N* = 814)	PCI (*N* = 832)	HR (95% CI)		
*At 30 days*									
MACCE	4.8 (4)	7.0 (5)	0.66 (0.18–2.46)	0.538	4.6 (37)	5.8 (48)	0.79 (0.51–1.21)	0.282	0.798
All-cause death, stroke, MI	4.8 (4)	7.0 (5)	0.66 (0.18–2.46)	0.538	3.7 (30)	4.5 (37)	0.83 (0.52–1.35)	0.460	0.745
All-cause death	2.4 (2)	4.2 (3)	0.56 (0.09–3.34)	0.523	0.5 (4)	1.9 (16)	0.26 (0.09–0.77)	0.015	0.466
Cardiac death	2.4 (2)	4.2 (3)	0.56 (0.09–3.34)	0.523	0.5 (4)	1.9 (16)	0.26 (0.09–0.77)	0.015	0.466
Any MI	3.6 (3)	5.7 (4)	0.62 (0.14–2.78)	0.534	2.2 (18)	3.6 (30)	0.61 (0.34–1.1)	0.102	0.994
Any stroke	0 (0)	0 (0)	0 (0–0)		1.4 (11)	0.1 (1)	11.35 (1.47–87.95)	0.020	0.998
Any repeat revascularization	1.2 (1)	2.8 (2)	0.42 (0.04–4.63)	0.478	1.4 (11)	3.1 (26)	0.43 (0.21–0.87)	0.020	0.977
*At 5 years*									
MACCE	35.9 (28)	47.3 (33)	0.73 (0.44–1.2)	0.213	27.1 (201)	36.8 (299)	0.67 (0.56–0.8)	< 0.001	0.805
All-cause death, stroke, MI	28.3 (22)	31.5 (22)	0.9 (0.5–1.63)	0.729	16.6 (121)	19.9 (163)	0.78 (0.62–0.99)	0.038	0.677
All-cause death	23.3 (18)	23.1 (16)	1.01 (0.52–1.99)	0.969	11.2 (79)	13.1 (107)	0.78 (0.58–1.04)	0.092	0.475
Cardiac death	11.7 (9)	16.1 (11)	0.73 (0.3–1.76)	0.483	5.0 (35)	8.4 (67)	0.55 (0.37–0.83)	0.004	0.563
Any MI	4.9 (4)	15.3 (10)	0.35 (0.11–1.11)	0.073	3.7 (29)	9.2 (73)	0.41 (0.27–0.63)	< 0.001	0.782
Any stroke	5.6 (4)	3.1 (2)	1.84 (0.34–10.04)	0.482	3.6 (27)	2.3 (18)	1.6 (0.88–2.9)	0.125	0.888
Any repeat revascularization	12.9 (9)	31.9 (20)	0.38 (0.17–0.84)	0.016	13.8 (101)	26.3 (202)	0.49 (0.39–0.63)	< 0.001	0.532
*At 10 years*									
All-cause death	42.3 (34)	43.9 (30)	0.96 (0.59–1.56)	0.858	22.7 (178)	27.1 (218)	0.81 (0.67–0.99)	0.041	0.554

*COPD* chronic obstructive pulmonary disease, *MI* myocardial infarction, *MACCE* major adverse cardiovascular and cerebrovascular event. Data are presented as percentage based on Kaplan–Meier estimates (number of deaths)

### Multivariable analysis

After adjustment for the baseline confounders, COPD remained an independent predictor of all-cause death at 10 years in the CABG arm (adjusted HR: 2.10, 95% CI: 1.19–3.69, *p* = 0.010), but was not an independent predictor in the PCI arm (adjusted HR: 1.19, 95% CI: 0.69–2.06, *p* = 0.536) (Table 5).

**Table 5:** The association between COPD and 10-year all-cause mortality

	Overall population		PCI arm		CABG arm	
	Adjusted HR (95% CI)	*p*	Adjusted HR (95% CI)	*p*	Adjusted HR (95% CI)	*p*
PVD	2.33 (1.71–3.17)	< 0.001	2.61 (1.64–4.16)	< 0.001	2.28 (1.48–3.51)	< 0.001
Current smoking	2.17 (1.59–2.97)	< 0.001	2.44 (1.60–3.73)	< 0.001	2.05 (1.28–3.30)	0.003
Age (per 10 years increase)	2.02 (1.67–2.43)	< 0.001	1.65 (1.29–2.11)	< 0.001	2.72 (2.01–3.69)	< 0.001
Previous stroke	1.72 (1.05–2.83)	0.033	1.20 (0.55–2.59)	0.645	2.33 (1.19–4.57)	0.014
Pharmacologically-treated diabetes	1.63 (1.25–2.12)	< 0.001	1.66 (1.16–2.38)	0.006	1.68 (1.14–2.49)	0.010
COPD	1.50 (1.01–2.20)	0.042	1.19 (0.69–2.06)	0.536	2.10 (1.19–3.69)	0.010
LVEF (per 10% decrease)	1.14 (1.03–1.25)	0.011	1.18 (1.02–1.33)	0.020	1.11 (0.96–1.28)	0.165
Previous MI	1.12 (0.86–1.47)	0.403	1.10 (0.76–1.59)	0.619	1.05 (0.69–1.61)	0.812
Anatomical SYNTAX score (per 10 point increase)	1.10 (0.99–1.21)	0.076	1.18 (1.03–1.35)	0.014	0.99 (0.84–1.16)	0.887
Female	1.04 (0.78–1.38)	0.795	1.07 (0.73–1.57)	0.740	0.97 (0.63–1.49)	0.882
Creatinine clearance (per 10 ml/min decrease)	1.03 (0.97–1.10)	0.273	1.09 (1.00–1.18)	0.055	0.97 (0.88–1.08)	0.567
Body mass index (per unit increase)	1.02 (0.99–1.05)	0.275	1.02 (0.98–1.06)	0.365	1.00 (0.95–1.05)	0.964

*COPD* chronic obstructive pulmonary disease, *LVEF* left ventricular ejection fraction, *MI* myocardial infarction, *PVD* peripheral vascular disease

## Discussion

To our knowledge, our study is the first analysis to evaluate the treatment effect of CABG versus PCI on 10-year all-cause mortality according to COPD in patients with 3VD and/or LMCAD. The main findings of the present study are summarized as follows:

1. No significant difference was found between COPD and non-COPD patients for 30-day MACCE. However, COPD was associated with a significantly increased risk of 5-year and 10-year all-cause death.
2. The relative treatment effects of CABG versus PCI on 10-year all-cause death were not significantly different for patients with COPD and patients without COPD.
3. COPD was an independent predictor of 10-year all-cause death after CABG but not after PCI.

### The impact of COPD on mortality after revascularization

COPD has been identified as a risk factor for worse clinical outcomes in CAD patients [3, 25]. However, the literature is inconsistent regarding the impact of COPD on mortality after revascularization. Angouras et al. found that COPD was not an independent predictor of increased early mortality, but was a continuing detrimental risk factor for long-term survival (mean follow-up, 7.6 years) in patients who underwent isolated CABG [26]. Analysis from SWEDEHEART registry demonstrated that patients with COPD had a significantly higher rate of both 30-day and 5-year mortality after CABG [15]. In patients who underwent PCI, previous reports showed discrepant results regarding the impact of COPD on short-term outcomes. Selvaraj et al. found that COPD was a significant independent predictor of in-hospital death and long-term mortality after PCI [16], while Berger et al. reported that in-hospital major adverse cardiac outcomes were not different between COPD and non-COPD groups. However, at 3-year follow-up, mortality for patients with COPD was significantly higher compared to those without COPD (21% vs. 9%, *p* < 0.001). The investigators found that COPD was independently associated with a twofold increase in the hazard of long-term mortality [27]. In our analysis, we observed that at 30 days patients with COPD had a comparable MACCE rate and a trend for a higher all-cause death (3.3% vs. 1.2%, HR: 2.66, 95% CI: 1.00–7.09, *p* = 0.050, Table 3). In terms of long-term survival, we found that COPD was associated with a significantly increased risk of 5-year and 10-year all-cause mortality both in the CABG arm and in the PCI arm. Our findings are consistent with previous studies reporting COPD is associated with a high risk of long-term mortality after revascularization [26, 27].

The explanations for these inconsistent results with respect to short-term mortality may be multifactorial. Varied definitions for COPD and different enrolled populations in these studies may partly contribute to the inconsistency. In addition, some studies have demonstrated that operative mortality after CABG is associated with the severity of COPD. A smaller study that evaluated the impact of COPD on CABG outcome found that only severe COPD influenced hospital deaths, so that hospital mortality in patients with mild-to-moderate COPD undergoing CABG was similar to those without COPD [28]. Subsequently, Fuster et al. found that in-hospital mortality was directly related to the severity of lung disease. Specifically, patients with forced expiratory volume in the first second (FEV1) < 60% predicted had higher mortality than those with FEV1 > 60%, and that this should be considered as a primary prognostic factor in COPD patients undergoing CABG procedures [29]. Moreover, restrictive lung disease may carry a greater prognostic impact than obstructive [11]. However, there are again inconsistent data. Michalopoulos et al. reported that patients with a history of mild or moderate COPD undergoing elective CABG had morbidity and mortality rates comparable to those without COPD [30]. Manganas et al. reported that the mortality rate associated with CABG is not affected by the presence and severity of airflow obstruction in patients with COPD, although the incidence of pulmonary infections and length of hospital stay were increased in patients with severe COPD [12]. Further studies are required to determine the impact of COPD on short-term outcomes.

### Is there an optimal revascularization strategy for patients with COPD?

To date, limited data exist in terms of the optimal revascularization strategy for patients with COPD and complex CAD. To our knowledge, our study is the first analysis to evaluate the treatment effect of CABG versus PCI on 10-year all-cause mortality according to COPD in patients with 3VD and/or LMCAD. In the EXCEL trial, patients with COPD had a trend for a higher all-cause death at 30 days (3.0% vs. 0.9%, *p* = 0.06) compared to those without COPD [18], which is similar to our findings. With regard to long-term outcomes, a higher 3-year mortality was observed in patients with COPD. Furthermore, in the EXCEL trial there were no statistically significant interactions in the relative risks of PCI versus CABG for the primary composite endpoint (death, stroke, MI or ischemia-driven revascularization) in patients with and without COPD at 3 years [18]. These findings are consistent with our current analysis, in which COPD was associated with a higher 10-year all-cause death, and no significant interaction between COPD and treatment strategy (CABG versus PCI) was found for all-cause death at 10 years. Although patients with COPD represent an increased surgical risk, patients with severe COPD have an acceptable long-term survival [11] compared to PCI and should therefore not be routinely denied CABG. More importantly, LIMA utilization in patients with COPD results in a significantly increased long-term survival, without an increased intensive care unit stay, re-intubation rate or in-hospital mortality rate [11]. Moreover, Ovaliet et al. found similar morbidity and mortality rates among the patients with and without COPD who underwent off-pump CABG [31]. Based on this evidence, CABG should be considered as an alternative revascularization strategy for patients with COPD. However, further large-scale studies are needed.

### COPD was an independent predictor of 10-year all-cause mortality after CABG but not after PCI

Previous studies had demonstrated that COPD was a predictor of long-term mortality after CABG [10]. A single-center analysis with a total of 10,994 patients found that COPD was a significant independent predictor of in-hospital death and long-term mortality after PCI [16], whereas another study reported that COPD was not an independent predictor of major adverse clinical outcomes in patients with STEMI following PCI [32]. In the SYNTAX trial, COPD was independently associated with 4-year mortality after CABG but not after PCI [4, 33]. Similarly, in SYNTAXES, with a follow-up out to 10 years, we found COPD was an independent predictor in the CABG arm but not in the PCI arm in the SYNTAXES. Therefore, COPD remains as one of the prognostic indexes in the SYNTAX score II 2020 [9]. COPD was a significant risk factor for non-adherence to medications, such as beta blockers, and underutilization of beta blockers in COPD was associated with clinicians concerns about bronchoconstriction [34]. In the SYNTAX study, patients with COPD were less likely to receive aspirin and beta blockers at discharge. However, after adjustment for the prescriptions of aspirin and beta blockers at discharge, COPD remains an independent predictor of 10-year all-cause death (HR: 1.52, 95% CI: 1.03–2.24, *p* = 0.037 in overall population, HR: 2.19, 95% CI: 1.24–3.89, *p* = 0.007 in CABG arm). Patients with COPD represent a high-risk cohort where adherence to treatment guidelines is crucial. Clinicians should take particular care with respect to the use of beta blockers and aspirin in patients with COPD, since prior studies demonstrated that both beta blockers and aspirin are not only safe but could also reduce all-cause death in patients with COPD [35, 36]. The majority of the studies in the field demonstrated that patients with a history of COPD have higher mortality rates than those without COPD, either after PCI or CABG. Therefore, in clinical practice, we should pay more attention to patients with COPD and use multidisciplinary care and self-management [37], a double health care approach that might improve the outcomes post revascularization no matter what modality of revascularization is used.

### Limitations

Several limitations should be considered in our current study. First, due to the modest sample size, the present analyses might not have adequate statistical power. Further large-scale trials more specifically dedicated to COPD patients are warranted. Second, this is a post hoc analysis and should be interpreted as hypothesis-generating only [38]. Third, the SYNTAX trial enrolled patients with de novo 3VD and/or LMCAD, and the findings should not be extrapolated to other CAD patients. COPD at baseline was defined according to the definition in EuroSCORE which is not specific for COPD; that definition does not differentiate asthma from COPD patients. However, it has been demonstrated in prior studies that asthma, as comorbidity, is independently associated with ischemic heart disease to an extent similar as COPD [39]. Another major limitation is that in the SYNTAXES study, the severity of COPD, emphysema, GOLD groups or grades, COPD exacerbations, pulmonary function tests (such as FEV1), and the use of long-term oxygen therapy or noninvasive ventilation were not available. Moreover, the endpoint in the SYNTAXES study was solely 10-year all-cause death. However, the SYNTAXES study provided data from the first randomized trial, comparing surgery and PCI with drug eluting stent, for which 10-year vital status was available in 93.8% of the patients. It has to be acknowledged that the PCI patients were treated with a first-generation drug eluting stent, which is no longer commercially available. Nevertheless, it is inevitable that any long-term observational data will be derived from a population treated with outdated/obsolete technology, while the evidence provided by contemporary technology can be derived only from short-term follow-up studies. Finally, an inherent bias may exist that patients with severe COPD who were deemed extremely high risk for CABG may not have been included in the randomized cohort and directed to the PCI registry in the SYNTAX trial.

## Conclusions

In the SYNTAXES trial, COPD was associated with a higher risk of 10-year all-cause death in patients with 3VD and/or LMCAD following either PCI or CABG. The risk of all-cause death at 10 years in patients with CABG versus PCI was similar irrespective of the prevalence of COPD.
